# Tissue-Engineered Tracheal Reconstruction

**DOI:** 10.3390/biomimetics10070457

**Published:** 2025-07-11

**Authors:** Se Hyun Yeou, Yoo Seob Shin

**Affiliations:** Department of Otorhinolaryngology-Head and Neck Surgery, Ajou University School of Medicine, Suwon 16499, Republic of Korea; entshyeou@gmail.com

**Keywords:** regenerative medicine, tracheal reconstruction, tissue engineering, scaffold, stem cells

## Abstract

Tracheal reconstruction remains a formidable clinical challenge, particularly for long-segment defects that are not amenable to standard surgical resection or primary anastomosis. Tissue engineering has emerged as a promising strategy for restoring the tracheal structure and function through the integration of biomaterials, stem cells, and bioactive molecules. This review provides a comprehensive overview of recent advances in tissue-engineered tracheal grafts, particularly in scaffold design, cellular sources, fabrication technologies, and early clinical experience. Innovations in biomaterial science, three-dimensional printing, and scaffold-free fabrication approaches have broadened the prospects for patient-specific airway reconstruction. However, persistent challenges, including incomplete epithelial regeneration and mechanical instability, have hindered its clinical translation. Future efforts should focus on the design of modular biomimetic scaffolds, the enhancement of immunomodulatory strategies, and preclinical validation using robust large animal models. Sustained interdisciplinary collaboration among surgical, engineering, and biological fields is crucial for advancing tissue-engineered tracheal grafts for routine clinical applications. Within this context, biomimetic approaches, including three-dimensional bioprinting, hybrid materials, and scaffold-free constructs, are gaining prominence as strategies to replicate the trachea’s native architecture and improve graft integration.

## 1. Introduction

Tissue engineering and regenerative medicine (TERM) is a multidisciplinary field dedicated to restoring, replacing, and enhancing the functions of tissues and organs damaged by diseases, trauma, or aging [1,2]. Through the integration of engineering, biological sciences, and clinical medicine, TERM facilitates the development of biocompatible tissue constructs ranging from cellularized scaffolds to whole artificial organs that emulate the structure and function of native human tissues. These constructs are typically generated by seeding autologous or allogeneic cells onto three-dimensional (3D) scaffolds composed of biomaterials followed by controlled stimulation and differentiation using bioactive molecules or bioreactor-based conditioning [3]. This approach provides a promising therapeutic alternative for cases where conventional surgical and pharmacological methods have proven insufficient, offering regenerative solutions tailored to patient-specific needs.

Tracheal regeneration has emerged as a particularly challenging application because of its unique anatomical, functional, and mechanical complexity. The loss of a long tracheal segment (>50% in adults and >30% in children) due to malignancy, trauma, prolonged intubation, or iatrogenic injury renders primary anastomosis unfeasible. Conventional approaches, such as metallic stents, silicone prostheses, and autologous tissue flaps, have achieved only partial success and are limited by complications including restenosis, infection, mechanical failure, and lack of integration [4]. Tissue engineering has attracted considerable interest as an alternative strategy for addressing these challenges [5,6].

Researchers have sought to reconstruct tracheal structures by implanting autologous cells onto biocompatible scaffolds and incorporating bioactive molecules to promote tissue maturation. Recently, scaffold technologies have advanced significantly, progressing from the use of decellularized donor tissues to patient-specific 3D-printed polymeric scaffolds [7,8]. Simultaneously, notable developments have been made in cell sources, such as induced pluripotent and mesenchymal stem cells, along with improvements in culture methods, including air–liquid interface (ALI) systems and bioreactor-based conditioning [9,10]. Although early studies demonstrated promising outcomes, several persistent challenges have hindered the clinical adoption of tissue-engineered tracheal grafts (TETGs). Mechanical weakness, insufficient vascularization and epithelialization, and immune rejection have played a role in the failure of previous clinical attempts [11].

Tissue-engineered tracheal reconstruction closely aligns with the principles of biomimetics, as it aims to replicate the native trachea’s structural, mechanical, and biological characteristics [12]. Although many existing scaffolds have yet to accurately reproduce the anisotropic cartilage framework and the flexibility of the posterior membranous wall, advancements in biomaterials and fabrication technologies are bringing these objectives closer to fruition [13]. Likewise, strategies for epithelialization and vascularization increasingly depend on mimicking the native cellular microenvironment [14]. These developments underscore a growing emphasis on biologically inspired design, which is fundamental to the field of biomimetics.

This review systematically summarizes the current progress in tissue-engineered tracheal reconstruction. We outlined the anatomical and physiological features of the native trachea, particularly the mechanical properties and respiratory epithelial characteristics that determine the scaffold design requirements. Various scaffold fabrication strategies have been examined, including the use of decellularized matrices, 3D printed constructs, electrospun nanofiber structures, and composite materials. Strategies for cellularization, such as the use of induced pluripotent stem cells, mesenchymal stem cells, ALI culture systems, bioreactor platforms, and cell-sheet techniques, have been reviewed with specific reference to preclinical and clinical outcomes. Current clinical experiences are discussed, existing limitations are assessed, and future directions necessary for the successful clinical translation of TETGs are proposed.

## 2. Normal Trachea Structure and Physiology

The trachea is a semi-rigid, flexible airway that provides a critical passage for air between the larynx and lungs [15]. Adults have a 10–13 cm long trachea with an internal diameter of approximately 2 cm. Structurally, the anterior and lateral aspects are reinforced by 18–22 C-shaped hyaline cartilage rings interconnected by elastic annular ligaments, whereas the posterior wall comprises smooth muscle fibers forming the tracheal muscle [16].

This composite structure ensures rigidity and flexibility and maintains airway patency against negative intrathoracic pressures while permitting controlled movement [6]. The luminal surface is lined with a pseudostratified ciliated columnar epithelium containing goblet cells, facilitating the mucociliary clearance of inhaled particles and pathogens. Beneath the epithelium lies the submucosa with mucus-secreting glands and connective tissue enveloped by the perichondrium, which surrounds each hyaline cartilage ring and is further supported externally by the adventitia, which anchors the trachea to the adjacent connective tissues [17]. Vascularization primarily arises from the branches of the inferior thyroid and bronchial arteries, whereas autonomic innervation regulates airway tone and reflexes [16,18].

### 2.1. Mechanical Properties

From a biomechanical standpoint, the native trachea must withstand dynamic intrathoracic pressure while maintaining its structural integrity and flexibility [19]. Achieving this balance is vital for normal respiratory function and poses a significant design challenge for tissue-engineered substitutes. Laterally, hyaline cartilage rings provide rigidity and prevent airway collapse during phases of high negative pressure, such as deep inspiration or vigorous coughing. Longitudinal flexibility is afforded by the posterior membranous wall, which comprises the tracheal muscle and elastic ligaments, allowing adaptation to cervical and thoracic motion without compromising airway patency [16]. Mechanically, the tracheal cartilage exhibits a biphasic viscoelastic behavior, combining solid-like stiffness with fluid-like damping properties under a mechanical load. In contrast, tracheal muscles exhibit hyperelastic characteristics, enabling significant reversible deformation under physiological conditions. Although mechanical properties vary based on sample preparation and testing methodologies, tracheal cartilage generally displays a high Young’s modulus, indicative of its role in resisting compressive forces [20].

**Figure 1:**
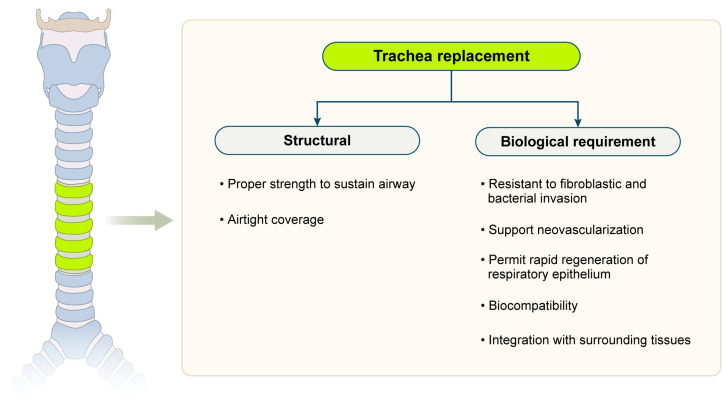
Design criteria for tissue-engineered substitutes.

Therefore, tissue-engineered grafts must meet the dual requirements of sufficient rigidity to prevent luminal collapse and sufficient flexibility to accommodate movement and growth. Failure to achieve this balance contributes to the collapse or dysfunction of early synthetic tracheal substitutes [21]. These mechanical requirements inform the essential design principles that must be addressed in engineering tracheal replacements (Figure 1) [22,23].

### 2.2. Respiratory Epithelium

**Figure 2:**
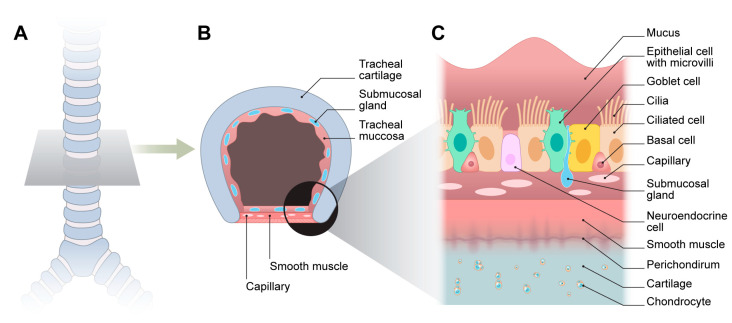
Anatomy of trachea. (**A**,**B**) Cross-section. (**C**) Cellular composition.

The respiratory epithelium lining the trachea is a specialized pseudostratified ciliated columnar tissue comprising ciliated, mucus-producing, goblet cells; basal progenitors; neuroendocrine cells; and epithelial cells with microvilli [24]. Along with the underlying submucosal glands, these epithelial components form a critical barrier that traps inhaled particulates and facilitates their clearance through the coordinated action of motile cilia (Figure 2).

Furthermore, the respiratory epithelium is integral to maintaining airway homeostasis and orchestrating immune responses. The rapid reestablishment of an intact epithelial barrier following injury or surgical intervention is essential to prevent infection, restenosis, and fibrotic remodeling [25]. Consequently, the successful regeneration of functional ciliated epithelium is a major objective of tissue-engineered tracheal reconstruction, directly affecting graft viability and long-term airway function [26].

## 3. Scaffold Strategies

Scaffold design for tracheal reconstruction must accommodate the unique biomechanical demands of the native airway. These include the anterior and lateral rigidity provided by the hyaline cartilage rings and the posterior flexibility afforded by the trachealis muscle and elastic connective tissue [6,16,20]. Although decellularized tracheal matrices inherently preserve this C-shaped anatomy [27], most synthetic and biofabricated scaffolds adopt circumferentially uniform tubular structures that may not fully replicate the anisotropic mechanics of the native trachea [7,28,29]. This discrepancy presents a critical challenge in designing structural and functional biomimetics [11].

### 3.1. Decellularized Trachea

**Figure 3:**
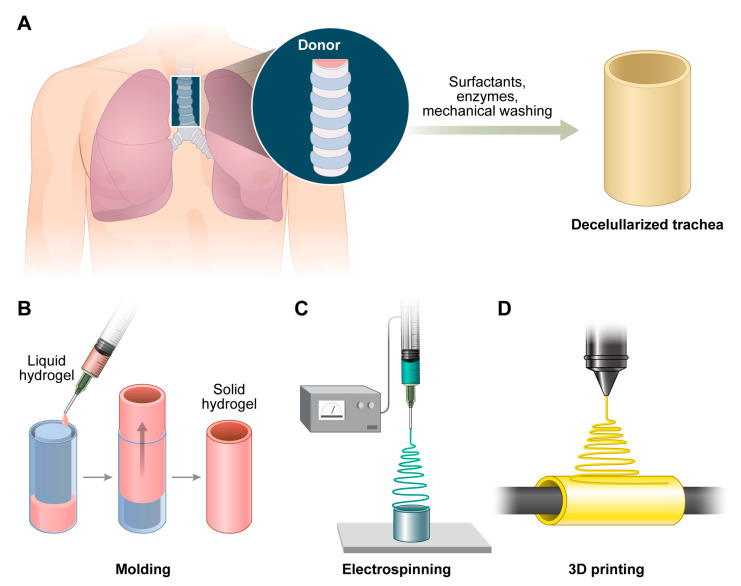
Common types of scaffolds. (**A**). Decellularized trachea. (**B**). Molding scaffolds. (**C**). Electrospun scaffolds. (**D**). Three-dimensional printed scaffolds.

Decellularized tracheal matrices are generated by removing all cellular components from the cadaveric or animal trachea using chemical agents, such as sodium dodecyl sulfate or Triton X-100; enzymatic digestion, such as DNase treatment; and/or physical techniques, such as freeze–thaw cycles [30] (Figure 3).

The decellularization of donor tracheae aims to eliminate immunogenic cellular components while preserving the structural and biochemical integrity of the extracellular matrix [27,30]. Retaining the native extracellular matrix is essential because it provides the three-dimensional architecture and molecular signals necessary for cellular adhesion, proliferation, and migration. Key matrix constituents such as collagen, elastin, and glycosaminoglycans, along with bound growth factors, play critical roles in guiding cell behavior and maintaining mechanical properties [27]. The preservation of the basement membrane and vascular conduits further facilitates epithelialization and revascularization after implantation. In contrast, overly aggressive decellularization protocols may disrupt the ultrastructure of the matrix, resulting in mechanical instability, reduced compliance, and even the collapse of the reconstructed airway. Notably, a substantial loss of glycosaminoglycan and elastin content has been associated with increased scaffold stiffness, which may impair physiological functions [27]. Therefore, optimizing decellularization techniques is necessary to balance effective cell removal with the maximal preservation of functional matrix elements [30].

Despite the reduction in antigenicity achieved through decellularization, residual immunogenicity remains a significant concern [31]. The incomplete removal of cellular remnants such as nuclear fragments, membrane debris, or species-specific epitopes can provoke host immune responses. To mitigate this risk, both quantitative and qualitative criteria have been proposed, including limiting the residual double-stranded DNA to <50 ng per milligram of dry matrix and ensuring that DNA fragments are <200 base pairs in length [27]. Compliance with these standards is associated with decreased immunogenic potential [32]. However, even when cellular material is adequately removed, matrix-bound epitopes intrinsic to xenogeneic tissues may still elicit immune activation. For instance, carbohydrate antigens naturally expressed in nonhuman sources can trigger antibody-mediated rejection if they are not specifically targeted during processing [27]. Furthermore, clinical experience has demonstrated that the implantation of unseeded acellular tracheal scaffolds may result in pronounced inflammatory reactions, fibrosis, or graft stenosis, ultimately compromising graft function [30]. To address these challenges, strategies such as pre-seeding decellularized matrices with autologous cells are increasingly being employed to promote host tolerance and improve integration [30,32].

### 3.2. Synthetic Scaffold

#### 3.2.1. Materials

Material selection for scaffolds serves as the foundation of any tissue-engineered cartilage construct. The key features of the representative scaffold materials used for tracheal reconstruction are summarized in Table 1 [28,29,33,34,35,36,37,38,39].

Natural scaffolds such as collagen, fibrin, and hyaluronic acid exhibit excellent biocompatibility, biodegradability, and cell adhesion properties; however, they often lack the mechanical integrity required for tracheal load-bearing applications [5]. In contrast, synthetic polymers such as polyglycolic acid, poly (lactic-co-glycolic acid), and polycaprolactone (PCL) offer superior mechanical strength and tunable degradation rates. In particular, PCL is U.S. Food and Drug Administration (FDA)-approved for certain clinical applications and is amenable to high-resolution 3D printing, making it a leading candidate for scaffold-based tracheal engineering [33,34]. To compensate for the limitations of single-material scaffolds, hybrid constructs combining synthetic polymers with natural extracellular matrix (ECM) components have been developed to enhance structural and biological performances.

#### 3.2.2. Molding/Electrospinning/3D-Printed Scaffolds

In addition to the choice of materials, the fabrication method plays a critical role in determining scaffold architecture, mechanical integrity, and clinical applicability. Commonly used approaches for tracheal scaffold fabrication include molding, electrospinning, and 3D printing (Figure 3). Traditional molding methods involve casting polymeric or hydrogel materials into tubular constructs using prefabricated molds [30]. Porosity is typically introduced by solvent casting, gas foaming, or salt leaching, which generates interconnected pores essential for cell infiltration and vascularization [40]. Molding has advantages in terms of simplicity, reproducibility, and scalability and is particularly suitable for generating standard geometries. However, mechanically reinforced components are often necessary because molded scaffolds may lack structural robustness and exhibit irregular porosity, which limits their endothelial ingrowth [30].

Electrospinning involves the use of high-voltage electrostatic forces to create nanofibrous mats that closely mimic the nanoscale architecture of the natural ECM [41]. Electrospun scaffolds fabricated from polymers, such as poly (l-lactic acid), PCL, and composite blends with collagen, promote cellular attachment and proliferation [42]. Nevertheless, creating mechanically stable, large-diameter tubular constructs remains difficult because of their insufficient intrinsic rigidity and limited pore interconnectivity, which impair vascular ingrowth *in vivo* [35]. Therefore, electrospinning is often used in combination with other reinforcement methods.

Three-dimensional printing technology allows for the layer-by-layer construction of patient-specific scaffolds with precise control over architectural features, including pore size, porosity, and mechanical properties [43]. This approach is suitable for producing patient-specific designs derived from imaging data [44]. PCL, polyurethane, and polylactic acid are frequently used because of their printability and biocompatibility. However, 3D printed scaffolds frequently require surface modifications, such as biomolecular coating, to enhance cellular adhesion and integration. Three-dimensional bioprinting has opened up additional possibilities for integrating microvascular channels directly into the scaffold design [45]. Although this technology has great theoretical potential, most studies remain at the proof-of-concept stage owing to limitations in resolution, mechanical integrity, and cell survival [37].

Each fabrication method has distinct advantages and limitations. Increasingly, hybrid approaches are being explored to combine these advantages and improve the scaffold performance for clinical translation.

#### 3.2.3. Others

Alternative scaffold-free methods have been investigated to overcome the drawbacks of synthetic scaffolds. These methods exploit the natural capacity of cells to self-assemble into 3D structures without relying on artificial matrices [46]. Techniques such as cell-sheet engineering, spheroid fusion, and tissue-strand fabrication focus on enhancing biocompatibility and minimizing immune responses by constructing grafts primarily from densely layered cellular sheets, including minimal self-secreted extracellular matrix components essential for cell–cell adhesion [39,47,48]. Although scaffold-free constructs have been shown to encourage biological integration, significant challenges persist, including limited mechanical strength, insufficient vascularization, and difficulties in scaling up for clinical application [39]. A comprehensive discussion of scaffold-free fabrication techniques and their respective advantages and limitations is presented in the Cell Strategy section.

## 4. Cell Strategy

### 4.1. Cell Types

Autologous chondrocytes and epithelial cells remain the gold standard for tracheal tissue engineering, owing to their lineage specificity and reduced immunogenicity. Mesenchymal stem cells (MSCs) derived from bone marrow or adipose tissue are attractive alternatives with immunomodulatory and multipotent properties [49]. Induced pluripotent stem cells provide an inexhaustible cell source but introduce concerns regarding genomic stability, tumorigenicity, and ethical considerations [50].

### 4.2. ALI Culture

The ALI culture plays a crucial role in tracheal tissue engineering by promoting the differentiation of basal airway cells into structurally and functionally mature mucociliary epithelia. When respiratory epithelial cells from the nasal cavity and bronchi are cultured on porous membranes with their apical surfaces exposed to air, they undergo extensive mucociliary differentiation and replicate the key features of the *in vivo* airway lining, including specialized cellular morphology and barrier integrity [51]. Pezzulo et al. demonstrated that primary human airway epithelial cells cultured under ALI conditions exhibit gene-expression patterns that closely resemble those found in native airway tissues, underscoring the significance of ALI systems in maintaining physiological transcriptional profiles [52].

**Figure 4:**
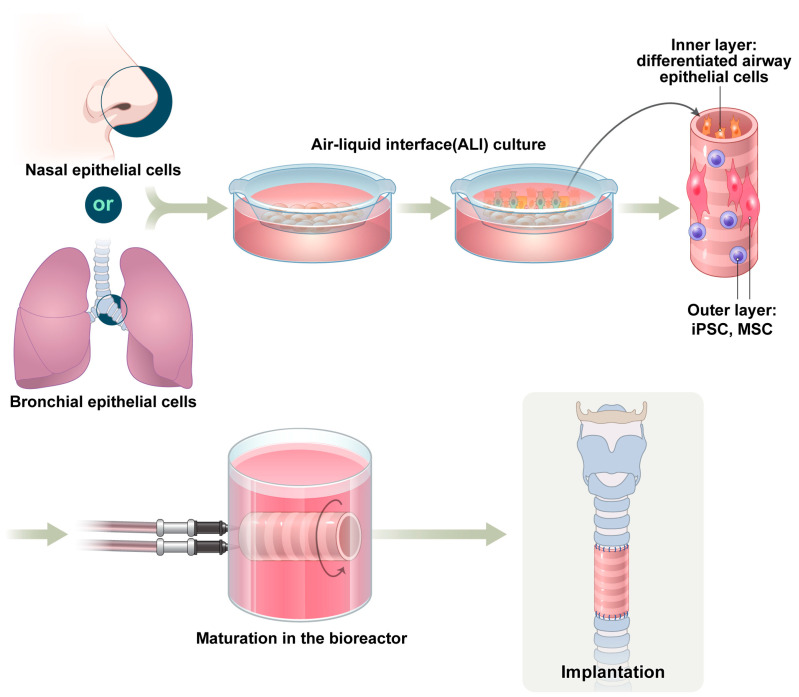
Air liquid interface (ALI) culture, cell seeding, and *in vitro* bioreactor technique. MSC, mesenchymal stem cell; iPSC, induced pluripotent stem cell.

Establishing a successful ALI culture requires the meticulous regulation of culture parameters. The basal medium must be optimized with essential biochemical components such as all-trans retinoic acid and carefully titrated growth factors. Recent advances in tracheal tissue engineering have integrated ALI systems with co-culture models that include stromal cells such as pulmonary fibroblasts and extracellular matrix analogs to support epithelial development [52]. The apical surface of the cell layer remains exposed to air, whereas the basal side receives nutrients through a permeable support membrane. This configuration promotes epithelial polarization and induces stratification, resulting in the progressive development of a pseudostratified architecture populated with basal, ciliated, and goblet cells (Figure 4) [53]. Within 2–3 weeks of ALI exposure, basal progenitor cells differentiate into cell types that are essential for airway homeostasis. By approximately day 21, cells expressing markers such as acetylated tubulin and MUC5B were identified, indicating the presence of motile cilia and mucus-secreting goblet cells, respectively [54]. As differentiation progresses, tight junctions form between cells, establishing an epithelial barrier with high integrity similar to that of the *in vivo* airway mucosa. The development of this barrier is crucial for defending the respiratory tract against pathogens and inhaled particulates because it supports the coordinated function of ciliary motion and mucus clearance [55]. Therefore, ALI culture has become a crucial methodological component for generating engineered tracheal grafts with physiologically relevant mucociliary functionality [52].

### 4.3. Bioreactor Preconditioning

#### 4.3.1. *In Vitro* Bioreactor

Dynamic bioreactor systems replicate physiological mechanical forces, including cyclic stretching, shear stress, and compression, to enhance scaffold maturation before implantation (Figure 4) [56]. Mechanical stimuli influence cell morphology, proliferation, differentiation, and ECM deposition. In particular, bioreactor preconditioning has gained increasing recognition as a critical step in achieving the reliable long-term performance of engineered tracheal grafts. Rotating wall vessel bioreactors and perfusion bioreactors are widely used in airway tissue engineering. Rotating wall vessels create a low-shear, microgravity-like environment that is ideal for maintaining cell aggregates and minimizing mechanical stress. By contrast, perfusion bioreactors provide a continuous flow of media through the scaffold, enhancing nutrient transport, oxygenation, and mechanical stimulation of cells within dense or thick tissues. Both systems have been successfully used to stimulate chondrogenic differentiation, support epithelial cell barrier formation, and promote uniform tissue development [57].

*In vitro* perfusion systems provide finely tunable control over environmental parameters such as pH, dissolved oxygen, flow velocity, and pressure [58]. This capability allows for the region-specific seeding of various cell types within tubular constructs [59]. For instance, in PEOT/PBT-based scaffolds, MSCs were seeded on the outer surface and subsequently differentiated into chondrocytes, whereas respiratory epithelial cells were seeded on the inner lumen [60]. Additional evidence supports the use of decellularized intestinal matrices seeded with progenitor cells, including MSCs, respiratory epithelial cells, and endothelial progenitors, to create hybrid tracheal grafts in perfusion bioreactors; however, the biomechanical and functional outcomes remain suboptimal for clinical applications [19]. More recently, dual-chamber rotating perfusion bioreactors have facilitated the simultaneous culture of MSCs and tracheal epithelial cells on decellularized tracheal scaffolds, resulting in enhanced cell attachment and uniform tissue organization compared to static conditions [9].

Such outcomes underscore the growing consensus that pre-implantation dynamic culture not only improves cellular outcomes and matrix maturation but also enhances the clinical viability of tracheal grafts by reducing ischemic injury and promoting early integration [61]. In this context, the combination of mechanically dynamic bioreactor culture and scaffold-specific cellular engineering is increasingly regarded as a rational and essential approach for the fabrication of functional airway substitutes [29].

#### 4.3.2. *In Vivo* Bioreactor

*In vivo* bioreactor techniques utilize the host body as a biological chamber, capitalizing on its native vascularity and immune privilege to facilitate preconditioning and tissue maturation. This approach minimizes the risk of contamination and enhances in situ vascularization, thereby effectively addressing the common limitations associated with prolonged *in vitro* culture. One study involved the application of human nasal septal chondrocytes embedded in a pluronic F127 hydrogel onto a trachea-shaped high-density polyethylene scaffold, which was subsequently implanted subcutaneously into athymic mice. The subcutaneous environment functioned as an *in vivo* bioreactor, resulting in >80% formation of cartilage-like tissue within 8 weeks, with no signs of inflammation [62]. Another study employed PGA or polypropylene mesh scaffolds seeded with epithelial and chondrocyte components, leading to the development of cylindrical constructs featuring pseudostratified epithelium and cartilage comparable to the native trachea after subcutaneous implantation in animal models [38]. An alternative study integrated cell self-aggregation technology with an in-body tissue architecture (iBTA) strategy to fabricate scaffold-free tracheal constructs. In this method, cartilage rings derived from rat chondrocyte monolayers were assembled around a central rod and implanted subcutaneously, resulting in a vascularized, cartilage-lined airway after 4 weeks [63].

Taken together, both *in vitro* and *in vivo* bioreactor systems play complementary roles in tracheal regeneration. *In vitro* systems facilitate tightly regulated scaffold conditioning and cellular organization, whereas *in vivo* strategies provide a biologically integrated pathway for vascularization and immunological adaptation. A synergistic approach that combines both techniques may be the most effective strategy for the clinical translation of TETGs.

### 4.4. Cell-Sheet/Spheroid Fusion/Tissue-Strand Techniques

#### 4.4.1. Cell-Sheet Technique

The cell-sheet technique facilitates the fabrication of scaffold-free continuous monolayers composed of epithelial or chondrocyte cells. These sheets can be stacked to create tubular constructs that mimic the layered architecture of native trachea. Temperature-responsive culture surfaces, particularly those coated with poly (N-isopropyl acrylamide), enable the non-enzymatic detachment of intact sheets by simply lowering the culture temperature. This method preserves the intercellular junctions and extracellular matrix components, both of which are critical for tissue integration after transplantation [64]. In addition to thermoresponsive platforms, other stimuli-responsive surfaces that react to light, pH, or electrical signals have been explored to expand the range of control over the detachment conditions [65,66]. Kanzaki et al. demonstrated that epithelial sheets transplanted onto Dacron scaffolds regenerated a pseudostratified ciliated epithelium within 4 weeks [48]. Chondrocyte-derived sheets can be cultured and concentrically layered to form cartilage-like laminae that replicate the anterior tracheal framework. When combined into bilayered constructs, these sheets provide a biologically derived conduit containing both mucosal and structural elements. However, their thin configuration and lack of vascularization limit their viability. To enhance graft survival and mechanical stability, researchers have investigated approaches, such as incorporating angiogenic factors, co-culturing with endothelial cells, and applying temporary structural supports [61].

#### 4.4.2. Spheroid Fusion Technique

Spheroid fusion involves the assembly of multicellular aggregates into predetermined 3D architectures using techniques such as bio-3D printing or micromanipulation. This approach enables spatial patterning of spheroids composed of various cell types, including basal epithelial cells, fibroblasts, MSCs, and chondrocytes, to replicate the region-specific organization of the trachea [67]. For example, cartilage spheroids may be arranged along the anterior portion of the construct, whereas fibroblasts or smooth muscle spheroids are positioned in the posterior segment [68]. As these spheroids mature in a bioreactor environment, they fuse to form a continuous tissue structure while preserving their cell-type-specific characteristics. These constructs demonstrated promising outcomes *in vivo*, including epithelialization, cartilage matrix deposition, and vascular ingrowth [39]. Temporary stenting is often used to maintain airway patency during the early remodeling phase. By avoiding synthetic materials, this approach minimizes the risks associated with chronic inflammation and foreign body response [44]. Notwithstanding these advantages, this study has notable limitations. Producing uniform and viable spheroids is labor-intensive, and prior to complete fusion, the constructs lack sufficient mechanical strength for surgical manipulation. To address these challenges, recent studies have investigated methods such as dynamic bioreactor conditioning, the co-delivery of matrix components, and controlled fusion timing [69].

#### 4.4.3. Tissue-Strand Technique

Tissue-strand fabrication generates elongated, cell-dense cords through self-assembly within tubular molds [70]. These strands can be aligned or braided to replicate the anisotropic structure of the tracheal wall [71]. Compared with spheroids, tissue strands offer improved initial cohesiveness and are easier to handle during the assembly of large constructs. Chondrocyte-loaded strands cultured under optimized *in vitro* conditions demonstrated robust extracellular matrix formation and mechanical performance, surpassing those of many hydrogel-based systems. This makes them particularly suitable for recreating both the rigid anterior cartilage and the more flexible membranous posterior portions of the trachea. Additionally, the strand format allows for the rapid scale-up of larger grafts. However, increasing the thickness of the strand-based constructs introduces diffusion-related constraints that compromise cell viability and tissue maturation [70]. To address these limitations, researchers have employed bioreactors that are capable of providing active perfusion and mechanical stimulation. These systems enhance nutrient transport, promote chondrogenic or myogenic differentiation, and facilitate early vascular network formation. Co-culture strategies involving endothelial or mesenchymal stromal cells are also being explored to further improve integration and long-term survival after implantation [39].

## 5. Clinical Application

Despite substantial research over the past two decades, clinical application of TETGs remains challenging. To date, only a limited number of studies have involved the transplantation of engineered tracheal constructs into human patients, and no standardized product or surgical protocol has been established. Nevertheless, early experiences have provided critical lessons that will continue to shape future development. In 2005, Omori et al. reported the fabrication of a tracheal graft comprising a polypropylene mesh (Marlex; Ethicon Inc., Somerville, NJ, USA) coated with collagen and internally reinforced with a spiral polypropylene ring, which was successfully used to reconstruct a partial tracheal defect resulting from thyroid cancer invasion [72]. A 2008 clinical study demonstrated that the transplantation of a decellularized donor trachea reseeded the autologous bone-marrow-derived MSCs and bronchial epithelial cells of the recipient without the need for long-term immunosuppression. Postoperative assessments demonstrated preserved airway patency, epithelialization, cartilage viability, and restored respiratory functions. However, subsequent attempts by Macchiarini et al. to use synthetic scaffolds in multiple patients led to serious complications, including scaffold collapse, fistula formation, and patient death. Many of these procedures have been performed without an adequate preclinical validation of scaffold functionality or cell-seeding effectiveness, raising significant ethical and scientific concerns [73]. This controversy has profoundly affected the field, highlighting the critical need for rigorous standards, robust preclinical evidence, and strict ethical oversight of clinical translation.

In 2010, Elliott et al. successfully transplanted a decellularized trachea reseeded with autologous MSCs and epithelial cells into a 10-year-old child with critical airway stenosis. Vascularization was promoted using a surgically fashioned omental flap, and the patient demonstrated sustained airway patency and normal growth over a 2-year follow-up period [49]. However, at 4 years post-transplantation, the graft showed insufficient growth relative to the development of the patient, along with complications such as restenosis and infections, requiring multiple additional interventions [74]. These outcomes underscore the need for enhanced vascularization, mechanical stability, and epithelial regeneration in tissue-engineered tracheal constructs. In 2013, Zopf et al. reported the first clinical application of a 3D printed bioresorbable airway splint in infants with severe tracheobronchomalacia. Using patient-specific imaging data, they fabricated a PCL-based splint that was surgically implanted to stabilize the airway, resulting in the immediate resolution of airway collapse and successful recovery. Although the splint serves as an external support rather than as a true tracheal replacement, this case demonstrates the potential to expand the clinical applications of tissue-engineering technologies.

Several clinical trials have evaluated the safety and efficacy of TETGs in humans. Table 2 summarizes the key trials, detailing their designs, investigational approaches, target populations, and preliminary clinical outcomes [75,76,77,78]. These efforts represent a critical step toward translating TETG concepts into clinical practice. However, the clinical application of TETGs remains limited to compassionate-use cases and investigational settings under emergency conditions. Long-term outcome data are sparse, and, to date, no tissue-engineered tracheal products have achieved widespread regulatory approval [28]. Regulatory bodies, including the FDA, mandate rigorous preclinical validation before authorizing clinical trials. This includes the demonstration of adequate vascularization, epithelialization, and mechanical integrity in large animal models. Although large animal models, such as pigs and dogs, are commonly used for preclinical validation, it is crucial to acknowledge the significant anatomical and biomechanical differences between human and quadrupedal tracheae [79]. In humans, the trachea is oriented vertically and experiences gravitational forces along its axis, whereas in quadrupeds, the trachea is positioned horizontally, which alters the distribution of mechanical stress during respiration [80]. Furthermore, most quadrupeds possess nearly complete cartilaginous rings or elliptical ring structures, in contrast to the C-shaped hyaline cartilage with a posterior membranous wall found in humans [81,82]. These differences impact airway compliance, mucus clearance, and the response to changes in intrathoracic pressure, potentially limiting the translational accuracy of graft performance when transitioning from animal studies to human clinical applications [80,83]. Consequently, these factors represent a substantial hurdle to the routine clinical adoption of TETG platforms.

The accumulation of clinical experience yielded several key findings. First, a systematic preclinical validation process that sequentially advances from small to large animal models is critical [84]. Second, the development of effective grafts requires interdisciplinary collaboration among the surgical, engineering, and biological fields. Third, surgical techniques such as the use of vascularized flaps, tension-free anastomoses, and temporary airway support devices significantly affect graft integration and survival [74,85]. Finally, rigorous postoperative monitoring through bronchoscopy, pulmonary function testing, and imaging studies is essential for the early detection of complications and the achievement of long-term success [74]. Overall, these experiences emphasize the necessity for cautious clinical translation supported by robust scientific evidence and strict ethical oversight.

## 6. Conclusions

Thus, TETGs hold great promise as functional airway substitutes for long-segment tracheal defects. Over the past two decades, significant progress has been made in scaffold design, cell sourcing, and preclinical testing. However, persistent challenges, particularly in achieving synchronous chondrogenesis, epithelialization, and vascularization, have limited their clinical translation.

Future research should prioritize the development of modular biomimetic scaffolds that enable spatial control of cell differentiation and mechanical support. Advances in bioprinting, immunomodulatory materials, and patient-specific design algorithms may bridge the gap between laboratory protocols and clinical applications. Standardized animal models, long-term functional evaluations, and rigorous clinical trials are essential to validate safety and efficacy. Ultimately, the convergence of bioengineering, stem-cell biology, and surgical innovation will determine whether tissue-engineered tracheas can transition from experimental constructs to routine clinical therapies.

## Figures and Tables

**Table 1:** Detailed comparison of scaffold materials for tracheal tissue engineering.

Scaffold Type	Material Example	Biological and Mechanical Properties	Limitations	Clinical/Preclinical Use
Natural	Collagen	· Excellent biocompatibility · Supports cell adhesion · Weak mechanical strength · Not load-bearing	· Rapid degradation · Weak for tracheal load	Used in early-stage research and animal models [28,34]
	Fibrin	· Supports angiogenesis · Biodegradable · Poor structural support under compression	· Difficult to maintain the tubular shape	Tested for short-segment tracheal repair [28]
	Hyaluronic Acid	· Extracellular matrix (ECM) mimicry · Promotes wound healing · Low strength · Fast degradation	· Requires crosslinking or hybridization	Tested in composite scaffolds using animal models [37,38]
Synthetic	PCL (Polycaprolactone)	· Biocompatible · U.S. Food and Drug Administration (FDA)-approved · Supports 3D printing · Good strength · Maintains airway patency	· May cause inflammation · Limited cell interaction	Used in pediatric airway stents and clinical trials [29,33,35]
	PLGA (Poly (lactic-co-glycolic acid))	· Controlled degradation rate · Used in drug delivery · Moderate strength · Tunable by composition	· Potential acidic byproducts during degradation	Preclinical animal studies ongoing [28,34,35]
Hybrid	PCL + ECM proteins	· Combines biological and mechanical advantages · Enhanced strength and flexibility	· Complex fabrication · Reproducibility issues	Promising in large- animal models, under study [29,34,35,36,39]

**Table 2:** Registered clinical trials of tissue-engineered tracheal grafts.

Trial	P.I./Institution	Purpose	Approach	Status	Outcomes
INSPIRE: Phase I Tracheal Replacement Using Seeded Decellularised Scaffold (NCT02949414) [75]	Birchall, M./ University College, London	Evaluate TETG safety in severe stenosis/malacia	Decellularized donor trachea + MSCs + biodegradable stent	Phase I started in 2016, suspended early due to complications	Initial mucosal recovery, high stenosis/granulation halted trial
TRITON-01: Airway Replacement with Stented Aortic Matrices (NCT04263129) [76]	Martinod, E./ Avicenne Hospital, Assistance Publique–Hôpitaux de Paris	Assess long-term airway graft integration	Cryo-decell aortic graft + external silicone stent (no recellularization)	Completed (2019–2022, 35 patients)	0% 30-day mortality; >80% R0 resection; integration with manageable issues
Patient-Customized Bioprinting Technology for Practical Regeneration of the Respiratory Tract (Trachea) (NCT06051747) [77]	Bae, J. S./ Catholic University of Korea, Seoul St. Mary’s Hospital	The pilot of patient-specific 3D bioprinted trachea	Three-dimensional printed scaffold with autologous chondrocytes/epithelial cells	Active (pilot Aug 2023); 6-mo follow-up completed	Good graft integration and patency at 6 months
Bioresorbable Airway Splint Pivotal Clinical Trial (NCT06406452) [78]	Green, G. E./ C.S. Mott Children’s Hospital, University of Michigan	Evaluate PCL bioresorbable airway splint in infants with severe TBM	Three-dimensional printed PCL external splint, custom-fit	Ongoing (2025–), 8-year follow-up planned in 35 pediatric patients	Compassionate-use cases showed functional restoration and safe biodegradation

TETG, tissue-engineered tracheal grafts; PCL, polycaprolactone; MSC, mesenchymal stem cell; TBM, tracheobronchomalacia.

## Data Availability

The data presented in this study are available on request from the corresponding author.
